# Breastfeeding and Early Childhood Dental Caries: Evidence from Birth Cohort Studies in Australia and Brazil

**DOI:** 10.3390/healthcare14060726

**Published:** 2026-03-12

**Authors:** Helena Silveira Schuch, Marcos Britto Correa, Jane A. Scott, Iná S. Santos, Andréa Dâmaso Bertoldi, Flavio Fernando Demarco, Diep Hong Ha

**Affiliations:** 1 School of Dentistry, The University of Queensland, Brisbane, QLD 4072, Australia; d.ha@uq.edu.au; 2 School of Dentistry, Federal University of Pelotas, Pelotas 96010-610, RS, Brazil; marcos.britto@ufpel.edu.br (M.B.C.); ffdemarco@gmail.com (F.F.D.); 3 School of Population Health, Curtin University, Bentley, WA 6102, Australia; jane.scott@curtin.edu.au; 4 Postgraduate Program in Epidemiology, Federal University of Pelotas, Pelotas 96010-610, RS, Brazil; inasantos.epi@gmail.com (I.S.S.); andreadamaso.epi@gmail.com (A.D.B.); 5 Post-Graduate Program in Health and Behavior, Catholic University of Pelotas, Pelotas 96015-560, RS, Brazil

**Keywords:** breastfeeding, dental caries, oral health, longitudinal studies

## Abstract

While breastfeeding is strongly recommended for infant health, uncertainty remains regarding its independent association with early childhood caries after accounting for sugar exposure. **Objective:** This study aimed to evaluate the controlled direct effect of breastfeeding on dental caries. **Methods:** Data were drawn from two population-based birth cohort studies: the 2015 Pelotas Birth Cohort Study (Brazil) and the SMILE Study (Australia). The exposure was any breastfeeding at 3, 6, and 12 months, with sugar consumption at 12 and 24 months as the mediator. The outcome was dental caries at ages 4/5, assessed as early childhood caries (ECC), severe ECC, any disease experience, or any decayed teeth. Marginal Structural Models with inverse probability of treatment weight were used to estimate the controlled direct effect of breastfeeding on dental caries, accounting for sociodemographic confounders and sugar consumption. **Results:** A total of 751 Australian and 3545 Brazilian children were included in at least one sub-analysis. Findings indicate a contextual difference: in Australia, breastfeeding had no effect on dental caries after considering sugar consumption as mediator, whereas in Brazil, children not breastfed had a lower risk of dental caries. For instance, adjusted Brazilian estimates showed a reduced relative risk ranging from 0.63 (95% CI 0.55; 0.72) for ECC to 0.43 (95% CI 0.34; 0.55) for severe ECC. **Conclusions:** The association between breastfeeding and dental caries appears to vary across socio-environmental contexts. In settings with high caries burden, prolonged breastfeeding may increase caries risk independently of sugar consumption; however, breastfeeding remains strongly recommended given its substantial overall health benefits. These findings highlight the importance of integrating early oral health guidance such as oral health education into breastfeeding support programs.

## 1. Introduction

Breastfeeding is widely recognised for its substantial short- and long-term health benefits for both infants and mothers and is strongly recommended by the World Health Organization [1,2,3,4,5]. However, in the context of oral health, the relationship between breastfeeding and dental caries remains complex [6]. Some studies have reported that sustained breastfeeding beyond 12 months may be associated with increased risk of early childhood caries (ECC) whereas breastfeeding for shorter durations has generally shown no association with ECC and may even be protective compared with formula feeding [7,8,9,10,11]. ECC is the most prevalent oral health problem affecting children, and it ranks first among all diseases concerning children aged 0 to 14 y [12]. Its potential impacts include dental pain, elevated rates of hospitalization and general anaesthesia, restricted social interaction, school absence, negative impacts on daily activities and the oral health-related quality of life, and financial burdens for the family and the health care systems [13]. ECC is also an important marker of dental caries later in life [14,15].

Diet is a key determinant of dental caries, with sugar widely recognized as a necessary cause of its development [16]. Breastfeeding has been linked to healthier eating behaviours and reduced food neophobia [17] which, in turn, is associated with lower consumption of sugar-rich ultra-processed foods [18]. Breastfeeding practices can influence caregiver feeding decisions, including when and how sugary foods and drinks are introduced. There is recent evidence on risk factors for early sugar introduction, and breastfeeding duration was an identified factor in both South America and Oceania, with the absence or shorter breastfeeding duration associated with early life sugar consumption [19]. Breastfeeding may also influence oral microbiota cariogenicity, as lactose accounts for about 40% of mature breast milk’s energy, though this remains unclear [5,9]. Early dietary practices, particularly sugar intake, may mediate the breastfeeding-caries relationship, though a direct effect independent of diet is also possible [20]. While systematic reviews have reported an association between sustained breastfeeding and ECC, they suggest this link is not yet fully established [7,8], and few studies have accounted for sugar intake in this relationship [21,22,23].

Other important limitations of the original studies reviewed include inadequate control for confounders and inconsistencies in variable measurement [7,8,9,11]. Since breastfeeding is culturally influenced [1,6], its association with dental caries may be context-dependent, including differences between high- and low-/middle-income countries. Most studies rely on cross-sectional designs and assess caries early, often at age two. Longitudinal studies tracking children through early years with detailed dietary data, particularly on sugar intake, are essential to clarify how breastfeeding at different stages affects dental caries over time [21].

This study examines the association between breastfeeding duration and early childhood caries (ECC) using data from two birth cohorts: the 2015 Pelotas Birth Cohort (Brazil) [24] and the SMILE Study (Australia) [25]. The study aims to assess the controlled direct effect of breastfeeding duration on ECC experience and severity, independent of sugar consumption and sociodemographic or family confounders.

## 2. Materials and Methods

### 2.1. Data Sources

#### 2.1.1. Australia: The Study of Mothers’ and Infants’ Life Events Affecting Oral Health (SMILE) Birth Cohort Study [25]

The SMILE study is an ongoing prospective birth cohort in Adelaide, South Australia (population ~1.3 M). Recruitment took place from July 2013 to August 2014 in the city’s three main public hospitals, covering about one-third of all births. Since not all births in the city were included in the eligible population, strategies were performed to recruit a population-representative sample by socioeconomic status. All new mothers in these hospitals were approached in the first 48 h after giving birth, and their newborn babies were invited to take part in the study. Mothers received dental care packages as incentives. Of the 2181 recruited mother-infant dyads, 96.8% (2112) completed the baseline questionnaire.

Follow-up data were collected when children were approximately 3, 6, 12, and 24 months of age, and again at 5 and 10 years. Data collection included caregiver-completed questionnaires, interviews, and physical assessments. Dietary information, including breastfeeding and sugar intake, was collected through caregiver questionnaires during early childhood follow-ups. Clinical oral examinations were conducted when children were 2, 5, and 10 years of age.

#### 2.1.2. Brazil: The 2015 Pelotas Birth Cohort Study [24]

In 2015, all hospital-born children, whose mothers lived in the urban area of Pelotas, South Brazil (population ~350,000), were identified and invited to participate in a cohort study. All mothers were approached and, out of the 4333 eligible live births, 4275 agreed to participate (response rate of 98.7%). Almost all of them answered the baseline questionnaire in the first 48 h after delivery (99.2%).

Participants were followed at approximately 3 months, 1 year, 2 years, and 4 years of age. Data collection included caregiver-completed questionnaires, interviews, cognitive assessments, and physical examinations. Information on children’s diet, including breastfeeding and sugar consumption, was obtained through caregiver questionnaires during early childhood follow-ups. Clinical oral examinations were conducted when children were 4 years old.

These two countries were included because they had comparable study designs, similar periods of data collection, and harmonised exposure and outcome measures, allowing for meaningful and methodologically robust cross-country comparison. All data collection was performed by trained and calibrated researchers and conducted in the official language of each country: English in Australia and Brazilian Portuguese in Brazil.

### 2.2. Variables of Interest

#### 2.2.1. Exposure

The exposure of interest was breastfeeding. Our primary exposure was any breastfeeding, as defined as still receiving breast milk, alone or in combination with other sources of food or drink. Therefore, any breastfeeding exposure included children to whom breast milk represented any proportion of their diet, comprising both those exclusively breastfed and partially breastfed. The exposure was evaluated as the practice at 3, 6, and 12 months and categorized as “yes” or “no” at each of these times.

We additionally evaluated exclusive breastfeeding. This information was available for Brazil at 3 and 6 months, and for Australia at 3 months. Exclusive breastfeeding was defined as “yes” if breast milk was the sole source of nutrition for the child. If the child reported having any other sources of food or drink, including water, they were identified as not being exclusively breastfed.

Breastfeeding was reported by the mother or first caregiver in each interview. At each time, they were asked whether the child was currently receiving any breastmilk. Upon a negative response, they were questioned how old the child was, in months and days, at weaning. Caregivers were also questioned about any other sources of food or drinks. This information was combined to estimate those who were still breastfed at the ages of interest and those exclusively breastfed.

#### 2.2.2. Mediator

Sugar consumption was assessed in both cohorts using parent-reported dietary information collected during follow-up visits and summarized into indicators at 12 and 24 months of age. Because the dietary assessment methods differed substantially between the Brazilian and Australian studies, direct comparison of absolute sugar intake was not feasible. Therefore, cohort-specific measures of sugar consumption were derived from the available dietary data and subsequently harmonized into comparable categorical indicators.

In the Brazilian study, sugar consumption was assessed using caregiver questionnaires. At 12 months, children were classified as consuming sugar if caregivers reported that the child consumed any of the following: sugar, honey, chocolate milk, soft drinks, or if sugar was added to bottled drinks. At 24 months, sugar consumption was considered present if caregivers reported intake of any of the following products: bottled, boxed, or powdered juice, bottled or boxed coconut water, soft drinks, sweet cookies, candies, lollipops, chewing gums, chocolates, jelly, and any sugar in the bottled drinks.

In the Australian study, dietary information was collected using different instruments at each age. At 12 months, sugar intake was estimated using a 24 h dietary recall via telephone interview together with a two-day estimated food record, covering two weekdays and one weekend day. All reported foods were entered into FoodWorks version 8 (Xyris Software, 2012–2017, Brisbane, Australia) and analysed using the Australian food composition database (AUSNUT 2011–13) [26], complemented with additional products included by the researchers [27]. At 24 months, sugar consumption was assessed using a food frequency questionnaire specifically developed for the SMILE Study (SMILE-FFQ) [28].

To facilitate cross-cohort analyses, sugar consumption was harmonized into binary variables within each cohort and age. At 12 months, children in Brazil were classified as consuming sugar if caregivers reported intake of any of the listed products. In Australia, sugar consumption was defined as >1 g of sugar intake based on dietary assessment, while ≤1 g was classified as no consumption.

At 24 months, a dichotomous definition was not feasible because the vast majority of children in both cohorts consumed some sort of sugar (only 5.3% of Brazilian parents reported no intake of sugary items and no Australian children had zero sugar intake). Therefore, sugar consumption at 24 months was categorized according to cohort-specific median distributions. In Brazil, children consuming up to 3 items from the list were classified as having lower consumption, while those consuming four or more items were classified as having higher consumption. In Australia, the classification was based on the median daily intake of sugar in grams (cut-off was 78.1 g).

Although the original dietary assessment methods differed between cohorts, both approaches provide valid indicators of sugar exposure in early childhood and have been used in previous studies [29]. Nevertheless, differences in dietary assessment instruments and food composition between countries limit direct comparability of absolute sugar intake.

#### 2.2.3. Outcome

The outcome was dental caries. Both prevalence and severity of the disease were assessed, as well as the presence of untreated disease. Considering the young age of the study population, dental caries evaluation included incipient markers of disease (i.e., white spots) and more severe, advanced stages of dental caries (i.e., from cavitated lesions).

Dental caries prevalence was categorized as ECC, severe ECC (S-ECC), any disease experience, or any decayed teeth. ECC was defined according to the WHO and the American Academy of Pediatric Dentistry as the presence of one or more decayed (noncavitated or cavitated lesions), missing (due to caries), or filled tooth surfaces in any primary tooth in a child under the age of six. S-ECC was defined as one or more cavitated, missing (due to caries), or filled smooth surfaces in primary maxillary anterior teeth or a decayed, missing, or filled score ≥ 5 at age 4 years (Brazil) or ≥ 6 at age 5 (Australia) [30]. Dental caries experience was measured as the decayed, missing, or filled surfaces (dmfs) score greater than 0, including only cavitated lesions in the decayed component. Finally, decayed teeth included only the ‘d’ component of the dmfs score, indicating children with cavitated caries lesions at the time of evaluation.

Dental caries was clinically measured by trained and calibrated dental professionals in both studies. Measurements were recorded at the surface level. Dentists used standard personal protective equipment, a headlamp, mouth mirror, and an NIDR (National Institute of Dental Research) periodontal probe. For the SMILE Study, examiners had an intraoral fiber optic light mouth mirror (MirrorLite), while a regular mouth mirror was available in the 2015 Pelotas Study. Studies had examinations conducted both at the Research Centres and at the family’s home. In Brazilian study, ECC was measured at 4 years of age; in the Australian study, at age 5. The outcomes of ECC and S-ECC are defined according to the child’s age.

#### 2.2.4. Confounding Factors

Potential confounding factors, defined as variables independently associated with both exposure and outcome, included considered in our analysis. These were maternal age in years, maternal education, marital status (single vs. de facto/married), parity (first child, one previous birth, or two or more previous births), and family income. All variables were collected at the time of the child’s birth.

Maternal education was measured differently across countries: seven categories in Australia and four in Brazil. To avoid loss of information during data harmonisation, maternal education was analysed using the original country-specific categories.

Income was collected in monthly minimum wages (MW) in Brazil and annual wages in Australia, and, for descriptive purposes, ranked into quartiles within each study.”

#### 2.2.5. Covariate

Since dental caries is a cumulative disease and outcomes were measured at different moments in the studies, we included the child’s age in months at the time of the examination as a covariate, to improve comparability between studies.

### 2.3. Statistical Analysis

All analyses were conducted in Stata, version 17. Descriptive analysis was performed, estimating frequencies (n (%)) and 95% Confidence Interval or means and standard deviations of the variables of interest. Inverse Probability of Treatment Weighting (IPTW) was employed to make the individuals exchangeable on the confounding factors. Marginal Structural Models (MSM) were applied to estimate the controlled direct effect (CDE) of breastfeeding on dental caries not mediated by sugar consumption or confounded by sociodemographic variables. In addition to the mediator, an interaction term between breastfeeding and sugar consumption was included in all models to account for any interaction effect. The controlled direct effect (CDE(m)) is expressed as the average change in the potential outcome if the mediator is uniformly set to a level (M = m) in the population where the treatment is changed from level (X = x) to (X = x*). In our case, if sugar was uniformly set to no (12 months) or low consumption (24 months).

Sensitivity analysis: To evaluate whether there were strongly relevant unmeasured confounding factors, E-values were computed. These values indicate how strong the association of the confounding factor with both exposure and outcome, conditional on the measured covariates, would need to be in order to change or eliminate the observed effect of the exposure on outcome [31].

## 3. Results

For each sub-analyses of breastfeeding duration and sugar consumption, a complete cases sample was generated. 751 Australian and 3545 Brazilian children were included in at least one of the sub-samples. For breastfeeding at 3 months and sugar at 12 months, samples included 631 children in Australia and 3081 in Brazil. For exposure at 6 months and mediator at 12 months, it comprised 619 Australian and 3101 Brazilian children. Finally, for breastfeeding at 12 months and sugar at 24 months, the complete case samples were 667 in Australia and 3503 in Brazil.

Table 1 presents the distribution of the participants’ sociodemographic characteristics and dental caries outcomes. On average, Australians were older than 5 years old when they had their dental evaluation, while Brazilian children were generally assessed before their 4th birthday. Other sociodemographic covariates were collected at birth. Australian mothers had a mean age of 30.7 years, and Brazilian mothers were 27.6 years old. The proportion of children who had siblings at baseline was similar between countries (~50%). The prevalence of ECC was 32.8% (95% CI 29.5; 36.2) in Australia and 37.1% (95% CI 35.5; 38.7) in Brazil. Although Brazilian children were younger at the dental assessment, they presented a higher burden of untreated dental caries. S-ECC, which has an age-specific case definition, presented the most pronounced disparity, with a prevalence of 10.6% (95% CI 8.6; 13.1) among Australian kids, while in Brazil it affected 21.0% (95% CI 19.7; 22.4) of children.

Table 2 and Supplementary Table S1 report the relative frequency of exposures and mediators, and the cross-tabulation of breastfeeding at different ages, sugar consumption, and dental caries outcomes. At 3 months, around 80% of children were still breastfed (80.3% in Australia and 77.8% in Brazil). This small gap between countries increased to more than 10% difference in the prevalence of breastfeeding at 6 months: 65.4% of Australian children were still breastfed at that age, compared to 54.7% of Brazilian children. This pattern reversed at 12 months, with a higher prevalence of sustained breastfeeding identified among Brazilian children (42.8% in Brazil and 34.3% in Australia). In Brazil, children breastfed (not necessarily exclusively) at 6 or 12 months had higher levels of all four dental caries outcomes than non-breastfed peers. A similar pattern was seen for breastfeeding at 3 months, though confidence intervals overlapped for ECC. In Australia, no associations were observed between breastfeeding and dental caries. Higher sugar consumption was consistently linked to more dental caries.

Table 3 and Table 4 and Supplementary Table S2 show the effects of any breastfeeding on dental caries before and after adjusting for confounders. The CDE of breastfeeding differs between Brazil and Australia. In Australia, breastfeeding had no effect on dental caries when controlling for sugar consumption. Adjusted estimates showed a CDE of not breastfeeding at 12 months on dental caries experience of 0.99 (95% CI: 0.61–1.60) when accounting for sugar intake at 24 months. Results were consistent across crude and adjusted analyses for breastfeeding at 3, 6, and 12 months, with sugar consumption as the mediator. In Brazil, children not breastfed had a lower risk of dental caries. Adjusted estimates showed a reduced risk of all caries indicators at 48 months among those not breastfed at 12 months, ranging from 0.63 (95% CI: 0.55–0.72) for ECC to 0.43 (95% CI: 0.34–0.55) for S-ECC (Table 4). Sensitivity analyses indicated that an unmeasured confounder would need a relative risk of 2.61 to fully explain the CDE of breastfeeding at 3 months on decayed teeth in Brazil.

Table 5 and Supplementary Table S3 present crude and adjusted analyses for not exclusively breastfeeding and dental caries, aligning with findings for any breastfeeding. In Australia, no association was found, while in Brazil, not exclusively breastfeeding appeared protective against dental caries after adjusting for sugar consumption and other covariates. For example, Brazilian children not exclusively breastfed at 3 months had a 40% lower risk of dental caries experience (dmfs > 0) (0.60 (95% CI: 0.47–0.77)) compared to exclusively breastfed peers.

## 4. Discussion

Breastfeeding is justifiably praised for its positive impacts on the baby, the mother, and their relationship. In oral health, however, there is still uncertainty and concern, backed by the inconclusive evidence on effects of breastfeeding on dental health. This study provides robust evidence from birth cohorts in Brazil and Australia on the association between breastfeeding duration and early childhood caries. While our findings do not resolve this debate, they highlight methodological challenges in studying this relationship. To our knowledge, this is the first study to combine breastfeeding data from two distinct countries, suggesting that the association between breastfeeding and dental caries may be context-dependent.

Our findings build on previous hypotheses [9] that breastfeeding’s effect on caries may vary by population context. Using a counterfactual approach, we estimated the direct effect of breastfeeding duration on caries experience and severity, controlling for sugar consumption and sociodemographic factors. In Brazil, children who were not breastfed at a given age had lower caries risk than those still being breastfed. In Australia, no association was observed in any of the 16 tested relationships (three durations of any breastfeeding, one duration of exclusive breastfeeding, and four caries indicators). While further studies are warranted to elucidate the context-dependent pattern of the breastfeeding-dental caries association, some arguments can be pondered.

One key factor is the differing oral health profiles of the two countries, including caries burden and access to dental care. As revealed by our descriptive results, Brazilian children experience higher levels of dental caries, particularly S-ECC. Both countries have mixed healthcare models, with public coverage, as well as private, fee-for-service options. This baseline oral health profile may have impacted our findings, as well as any systematic differences in access to preventive oral healthcare. Diet, especially sugar consumption, is another critical aspect. Our main exposure was any type of breastfeeding, and we did not evaluate complementary foods. It is plausible that other foods and drinks may have played a role, and we may be attributing to breastfeeding an effect that is mediated via complementary feeding, not assessed in this research study. For context, findings from the 2015 Pelotas Birth Cohort showed that children with prolonged breastfeeding (24 months or more) and high consumption of ultra-processed foods had higher prevalence and experience of dental caries, although no interaction effect was observed [28]. Nevertheless, in our study, the association remained when exclusive breastfeeding was examined, strengthening the robustness of our findings. Other aspects of feeding practices, such as bottle feeding and nocturnal feeding, may also play a role in the breastfeeding-dental caries association and should be further evaluated.

The methods used to estimate sugar consumption differed significantly between Brazil and Australia, which may have influenced our findings. Accurately measuring sugar intake, particularly free sugar, is inherently challenging. In the Australian cohort, the SMILE-FFQ was specifically developed for this purpose [28], whereas in Brazil, sugar consumption was estimated using a more limited list of foods and beverages, as described in Section 2. Additionally, the stricter classification in Australia—where consuming at least one gram of sugar at 12 months was coded as “yes” for sugar consumption—likely contributed to the high prevalence (89.1%) observed in that population. In contrast, sugar consumption in Brazil at the same age was reported at just under 52%. While these estimates are not directly comparable, they represent the best possible approach given the reliance on data already collected. Nonetheless, sugar intake in Brazil is likely underestimated, and the use of a binary variable may oversimplify the complexity of dietary patterns, potentially impacting our findings.

Differential social patterning of infant feeding practices and the social structuring of dental caries must be considered. Some argue that comparing breastfeeding effects across socially diverse contexts helps clarify whether its influence on cognitive development is direct or due to residual confounding [1]. Societal attitudes toward public breastfeeding also vary and may contribute to cross-country differences in breastfeeding duration [32]. Although our models accounted for a comprehensive set of confounders in both countries, residual confounding remains possible and may explain the differing findings for Brazil and Australia. This aligns with discrepancies between observational studies and randomized controlled trials, as the latter better control for unmeasured confounding. Additionally, contextual factors such as maternal ethnicity, cultural breastfeeding norms, and health behaviours play a role, as breastfeeding frequency and duration are culturally shaped [33]. Maternity leave policies also vary by country, affecting breastfeeding continuity. Moreover, understanding contextual disparities in dental caries requires considering community water fluoridation levels, given their preventive effect and interaction with free sugar intake and breastfeeding [34,35]. However, as our study was conducted in settings with optimal fluoridation, this factor did not influence our findings.

This investigation is based on two population-based cohort studies, and the prospective assessment from birth to early childhood is among the strengths of this study. Data were collected using validated instruments, including a comprehensive evaluation of sugar consumption and dental caries clinically assessed by trained and calibrated dentists. In addition, it comprises data from two socio-demographically distinct contexts, Brazil and Australia. Particularly for socially patterned practices and conditions, such as breastfeeding and dental caries, being able to draw evidence from these two settings provides robustness and reliability to the findings.

This study has several limitations. Harmonizing data across two independent cohorts was challenging, as some variables (e.g., exclusive breastfeeding at 6 months) were unavailable in one dataset, limiting direct comparability. These challenges highlight the need for coordinated data collection in future studies to standardize measures of breastfeeding and dental caries, as previously recommended [9,36]. Measurement of sugar intake also differed between studies, and dietary data were self-reported, introducing potential measurement error. Given diet is a key risk factor for oral and general health and dental caries is sugar-dependent, future data should rigorously and consistently capture sugar intake. Given the lack of standardised methods to assess cariogenic diet [37], sugar consumption may have been misclassified, and simplified measures may not fully capture the amount and frequency of intake, potentially attenuating associations.

Residual confounding remains possible despite adjustment for a comprehensive set of covariates, as is inherent to observational research and may partly explain discrepancies with randomized trials. Contextual factors should also be considered when interpreting the findings. Although community water fluoridation exists in both countries, differences in exposure patterns, coverage, and complementary preventive practices may have influenced baseline caries risk and contributed to the variation observed between cohorts. Finally, follow-up duration differed between studies, with Australian children assessed at a later age. Although age was adjusted for in all models, this difference may have introduced bias.

Future research should prioritise coordinated, cross-country cohort studies with harmonised measures of breastfeeding (including frequency, nocturnal feeding, exclusivity, and duration), complementary feeding, dental caries outcomes, and rigorous, quantitative assessment of free sugar intake to improve comparability and reduce misclassification. The application of advanced causal inference methods to address time-varying confounding and mediation is essential to determine whether associations are causal or context-dependent. Greater attention to intersectional and contextual determinants, such as socioeconomic conditions, cultural norms, and access to preventive care, will help explain heterogeneity across settings. In addition, mixed-methods and implementation research are needed to generate evidence on how oral health promotion can be effectively embedded within breastfeeding support frameworks, particularly in high-burden populations.

The objective of understanding the potential risks associated with breastfeeding should not be seen as a disbelief in its benefits. While its optimal duration concerning dental caries remains unclear, breastfeeding is a healthy practice that should be encouraged. Based on current evidence, promoting breastfeeding beyond the first year should be paired with oral health education and prevention to mitigate potential dental caries risks [11]. The 2024 World Breastfeeding Week, themed “Closing the gap: Breastfeeding support for all”, emphasizes the need for universal breastfeeding support to reduce health inequities and uphold maternal and infant rights. A transdisciplinary approach integrating general and oral health care would further strengthen this support.

## 5. Conclusions

In conclusion, our findings suggest that the association between breastfeeding and dental caries may be context-dependent. In some settings, it may be a risk factor for dental caries in early childhood not through sugar consumption. This ongoing debate questions whether breastfeeding impacts dental caries through a causal pathway or is related to social and cultural factors not yet accounted for in most observational studies [9]. Given all the benefits of breastfeeding to maternal and child health and wellbeing, our findings underscore the importance of integrating early oral health guidance, such as timely toothbrushing, fluoride exposure, and appropriate complementary feeding, into breastfeeding support programs, particularly in populations with high caries burden.

## Figures and Tables

**Table 1:** Description of the participants for each country.

	Australia			Brazil		
**Child age at follow-up (in months)**						
Mean (median)	64.9 (64)			44.9 (45)		
**Maternal age at child’s birth**						
Mean (median)	30.7 (30)			27.6 (27)		
	n	%	95% CI	n	%	95% CI
**Maternal education at child’s birth ^1^**						
Highest	129	17.2	14.6; 20.0	1084	30.6	29.1; 32.1
Medium-high	321	42.7	39.2; 46.3	1260	35.5	34.0; 37.1
Medium-low	176	23.4	20.5; 26.6	896	25.3	23.9; 26.7
Lowest	125	16.5	14.1; 19.5	305	8.6	7.7; 9.6
**Family income at child’s birth**						
1	91	12.1	10.0; 14.7	912	25.7	24.3; 27.2
2	243	32.4	29.1; 35.8	934	26.3	24.9; 27.8
3	243	32.4	29.1; 35.8	872	24.6	23.2; 26.0
4 (highest)	174	23.2	20.3; 26.3	827	23.3	22.0; 24.7
**Partner at child’s birth**						
Yes	719	95.7	94.0; 97.9	3050	86.0	84.9; 87.1
No	32	4.3	3.0; 6.0	495	14.0	12.9; 15.1
**Parity at child’s birth**						
1	372	49.5	46.0; 53.1	1759	49.6	48.0; 51.3
2+	379	50.5	46.9; 54.0	1786	50.4	48.7; 52.0
**Early childhood caries (ECC)**						
No	505	67.2	63.8; 70.5	2230	62.9	61.3; 64.5
Yes	246	32.8	29.5; 36.2	1315	37.1	35.5; 38.7
**Severe early childhood caries (S-ECC)**						
No	671	89.4	86.9; 91.4	2800	79.0	77.6; 80.3
Yes	80	10.6	8.6; 13.1	745	21.0	19.7; 22.4
**Dental caries prevalence (dmfs > 0)**						
No	574	76.4	73.2; 79.3	2600	73.3	71.9; 74.8
Yes	177	23.6	20.7; 26.7	945	26.7	25.2; 28.1
**Untreated dental caries (d > 0)**						
No	618	82.3	79.4; 84.9	2629	74.2	72.7; 75.6
Yes	133	17.7	15.1; 20.6	916	25.8	24.4; 27.3
**Total ^2^**	**751**	**100.0**		**3545**	**100.0**	

^1^ Maternal education: In Brazil, the categories are: 12 or more years (completed high school or more), 9 to 11 years, 5 to 8 years (primary education), and up to 4 years. In Australia, they are: postgraduate studies, some university or college/completed university or college, some vocational training (e.g., trade)/completed vocational training and some high school/completed high school. This 4-group categorization in Australia was adopted for descriptive purposes only. ^2^ Total number of children included in at least one of the sub-analyses.

**Table 2:** Distribution of the outcomes by exposures and mediators.

	Australia (Age 5 Years)					Brazil (Age 4 Years)				
	Overall	ECC Present	S-ECC Present	dmfs > 0	d > 0	Overall	ECC Present	S-ECC Present	dmfs > 0	d > 0
	%	%	%	%	%	%	%	%	%	%
Exclusive breastfeeding at 3 months										
No	44.2	34.0	8.5	23.6	17.9	54.6	35.8	18.9	23.9	23.4
Yes	55.8	32.2	12.2	23.7	17.2	45.4	38.6	23.4	29.9	28.8
Exclusive breastfeeding at 6 months										
No	-	-	-	-	-	87.0	38.0	21.5	26.9	26.4
Yes	-	-	-	-	-	13.0	33.0	18.7	26.4	24.2
Any breastfeeding at 3 months										
No	19.7	35.2	9.1	23.2	19.0	22.2	33.9	16.6	21.2	20.7
Yes	80.3	32.4	10.9	23.7	17.2	77.8	38.0	22.2	28.2	27.3
Any breastfeeding at 6 months										
No	34.6	29.5	6.2	19.9	14.9	45.3	32.6	15.9	20.2	19.7
Yes	65.4	34.0	12.7	25.4	19.1	54.7	40.8	25.2	32.0	30.9
Any breastfeeding at 12 months										
No	65.7	32.8	9.1	22.0	16.0	57.2	32.0	15.5	20.4	19.7
Yes	34.3	33.1	13.5	26.3	21.1	42.8	43.8	28.3	35.0	34.0
Sugar consumption at 12 months										
No	10.9	29.6	8.4	21.1	15.5	48.2	27.6	13.1	18.5	17.6
Yes	89.1	32.5	10.5	23.1	16.9	51.8	44.2	26.3	32.0	31.4
Sugar consumption at 24 months										
Low	52.2	29.3	8.3	19.5	14.1	55.0	30.4	15.5	20.6	19.7
High	47.8	36.0	11.9	27.3	21.6	45.0	45.2	27.6	34.0	33.2

ECC: Severe Early Childhood Caries. dmfs: decayed, missing, or filled surfaces. d: decayed surfaces.

**Table 3:** Crude analysis for the controlled direct effect of not breastfeeding on dental caries. Marginal structural models.

		Australia Age 5 Years	Brazil Age 4 Years
		RR (95% CI)	RR (95% CI)
**Exposure**	**Not BF 3 months**	**n = 631**	**n = 3081**
**Mediator**	**Sugar 12 months**		
ECC	CDE	0.90 (0.31; 2.61)	0.78 (0.63; 0.97)
S-ECC	CDE	1.14 (0.64; 2.03)	0.60 (0.41; 0.88)
dmfs > 0	CDE	0.40 (0.06; 2.86)	0.63 (0.47; 0.86)
d > 0	CDE	1.11 (0.71; 1.74)	0.63 (0.46; 0.86)
**Exposure**	**Not BF 6 months**	**n = 619**	**n = 3101**
**Mediator**	**Sugar 12 months**		
ECC	CDE	0.83 (0.32; 2.15)	0.73 (0.62; 0.87)
S-ECC	CDE	0.79 (0.09; 6.55)	0.54 (0.41; 0.72)
dmfs > 0	CDE	0.86 (0.27; 2.72)	0.57 (0.45; 0.72)
d > 0	CDE	0.35 (0.05; 2.54)	0.56 (0.44; 0.71)
**Exposure**	**Not BF 12 months**	**n = 667**	**n = 3503**
**Mediator**	**Sugar 24 months**		
ECC	CDE	1.16 (0.82; 1.66)	0.63 (0.55; 0.73)
S-ECC	CDE	1.37 (0.64; 2.93)	0.41 (0.33; 0.52)
dmfs > 0	CDE	0.94 (0.61; 1.47)	0.49 (0.41; 0.59)
d > 0	CDE	0.76 (0.45; 1.30)	0.48 (0.39; 0.57)

Adjusted for age at dental examination and including an interaction term between exposure and mediator. BF: Breastfeeding. RR: Relative Risk. 95% CI: 95% confidence interval. ECC: Early Childhood Caries. S-ECC: Severe Early Childhood Caries. dmfs: decayed, missing, or filled surfaces. d: decayed surfaces. CDE: Controlled direct effect.

**Table 4:** Adjusted analysis for the controlled direct effect of not breastfeeding on dental caries based on Marginal structural models, and sensitivity analyses based on adjusted estimates.

		Australia Age 5 Years		Brazil Age 4 Years	
		aRR (95% CI)	E-Value	aRR (95% CI)	E-Value
			aRR		aRR
Exposure	Not BF 3 months	n = 631		n = 3081	
Mediator	Sugar 12 months				
ECC	CDE	1.05 (0.33; 3.35)	1.28	0.77 (0.60; 0.98)	1.92
S-ECC	CDE	1.13 (0.59; 2.16)	1.51	0.62 (0.40; 0.96)	2.61
dmfs > 0	CDE	0.45 (0.06; 3.38)	3.87	0.62 (0.43; 0.88)	2.61
d > 0	CDE	0.89 (0.52; 1.53)	1.50	0.62 (0.43; 0.88)	2.61
Exposure	Not BF 6 months	n = 619		n = 3101	
Mediator	Sugar 12 months				
ECC	CDE	1.14 (0.34; 3.80)	1.54	0.69 (0.57; 0.83)	2.26
S-ECC	CDE	1.95 (0.09; 3.88)	3.31	0.56 (0.40; 0.77)	2.97
dmfs > 0	CDE	1.22 (0.30; 4.98)	1.74	0.55 (0.42; 0.71)	3.04
d > 0	CDE	0.26 (0.03; 2.28)	7.15	0.54 (0.42; 0.71)	3.11
Exposure	Not BF 12 months	n = 667		n = 3503	
Mediator	Sugar 24 months				
ECC	CDE	1.16 (0.79; 1.70)	1.59	0.63 (0.55; 0.72)	2.55
S-ECC	CDE	1.37 (0.59; 3.19)	2.08	0.43 (0.34; 0.55)	4.08
dmfs > 0	CDE	0.99 (0.61; 1.60)	1.11	0.50 (0.42; 0.61)	3.41
d > 0	CDE	0.76 (0.43; 1.35)	1.96	0.49 (0.40; 0.60)	3.50

Adjusted for maternal education, household income, partnership, maternal age, parity, and age at dental examination, and including an interaction term between exposure and mediator. BF: Breastfeeding. aRR: adjusted Relative Risk. 95% CI: 95% confidence interval. ECC: Early Childhood Caries. S-ECC: Severe Early Childhood Caries. dmfs: decayed, missing, or filled surfaces. d: decayed surfaces. CDE: Controlled direct effect.

**Table 5:** Crude and adjusted analysis for the controlled direct effect of not exclusively breastfeeding on dental caries, and sensitivity analyses based on adjusted estimates. Marginal structural models.

		**Australia** **Age 5 Years**		
		**Crude RR ^1^** **(95% CI)**	**Adjusted RR ^2^** **(95% CI)**	**E-Value**
Exposure	Not Exclusive BF 3 months	n = 631	n = 631	aRR
Mediator	Sugar 12 months			
ECC	CDE	2.06 (0.91; 4.65)	1.91 (0.75; 4.87)	3.23
S-ECC	CDE	3.91 (0.43; 35.60)	5.82 (0.62; 54.67)	11.12
dmfs > 0	CDE	1.84 (0.65; 5.22)	1.70 (0.54; 5.40)	2.79
d > 0	CDE	1.30 (0.35; 4.76)	0.94 (0.22; 3.93)	1.32
		**Brazil** **Age 4 Years**		
		**Crude RR ^1^** **(95% CI)**	**Adjusted RR ^2^** **(95% CI)**	**E-Value**
Exposure	Not Exclusive BF 3 months	n = 3075	n = 3075	aRR
Mediator	Sugar 12 months			
ECC	CDE	0.77 (0.66; 0.91)	0.74 (0.61; 0.89)	2.04
S-ECC	CDE	0.63 (0.48; 0.82)	0.64 (0.47; 0.86)	2.50
dmfs > 0	CDE	0.61 (0.49; 0.76)	0.60 (0.47; 0.77)	2.72
d > 0	CDE	0.61 (0.48; 0.76)	0.60 (0.47; 0.78)	2.72
Exposure	Not Exclusive BF 6 months	n = 3062	n = 3062	E-value
Mediator	Sugar 12 months			aRR
ECC	CDE	0.97 (0.78; 1.21)	0.72 (0.56; 0.93)	2.12
S-ECC	CDE	0.99 (0.70; 1.40)	0.70 (0.45; 1.10)	2.21
dmfs > 0	CDE	0.85 (0.65; 1.11)	0.56 (0.41; 0.77)	2.97
d > 0	CDE	0.91 (0.68; 1.20)	0.58 (0.42; 0.81)	2.84

^1^ Adjusted for age at dental examination and including an interaction term between exposure and mediator. ^2^ Adjusted for age at dental examination, maternal education, household income, partnership, maternal age, parity, and including an interaction term between exposure and mediator. BF: Breastfeeding. RR: Relative Risk. 95% CI: 95% confidence interval. ECC: Early Childhood Caries. S-ECC: Severe Early Childhood Caries. dmfs: decayed, missing, or filled surfaces. d: decayed surfaces. CDE: Controlled direct effect.

## Data Availability

The data that support the findings of this study are not publicly available due to data sharing agreements but are available from the data sharing committee upon request.

## Supplementary Materials

The following supporting information can be downloaded at: https://www.mdpi.com/article/10.3390/healthcare14060726/s1, Table S1. Distribution of the outcomes by exposures and mediators. Table S2. Adjusted analysis for the controlled direct effect of not breastfeeding on dental caries based on Marginal structural models, and sensitivity analyses based on adjusted estimates. Table S3. Crude and adjusted analysis for the controlled direct effect of not exclusively breastfeeding on dental caries, and sensitivity analyses based on adjusted estimates. Marginal structural models. 2015 Pelotas Birth Cohort Study.

## Abbreviations

The following abbreviations are used in this manuscript:

ECC	Early childhood caries
S-ECC	Severe early childhood caries
SMILE	The Study of Mothers’ and Infants’ Life Events Affecting Oral Health
CI	Confidence Interval
FFQ	Food frequency questionnaire
IPTW	Inverse Probability of Treatment Weighting
MSM	Marginal Structural Models
CDE	controlled direct effect
dmfs	Decayed, missing, or filled surfaces
d	Decayed surfaces
MW	Monthly minimum wages
RR	Relative risk
aRR	Adjusted relative risk
