# Supplementary material for: Breastfeeding and Early Childhood Dental Caries: Evidence from Birth Cohort Studies in Australia and Brazil
Source: Healthcare (Basel). 2026 Mar 12;14(6):726. doi: 10.3390/healthcare14060726 (PMC13027037; doi:10.3390/healthcare14060726)
Supplement: Supplementary file 1 [file healthcare-14-00726-s001.zip › healthcare-4178406-supplementary.pdf]

healthcare

MDPI

Supplementary tables:

Supplementary Table S1. Distribution of the outcomes by exposures and mediators.

	**Australia (age 5 years)**				
	Overall	ECC present	SECC present	dmfs>0	d>0
	% (95% CI)	% (95% CI)	% (95% CI)	% (95% CI)	% (95% CI)
Exclusive breastfeeding at 3 months					
No	44.2 (40.6; 47.9)	34.0 (29.0; 39.3)	8.5 (5.9; 12.1)	23.6 (19.2; 28.6)	17.9 (14.1; 22.5)
Yes	55.8 (52.1; 59.4)	32.2 (27.8; 36.9)	12.2 (9.3; 15.8)	23.7 (19.8; 28.1)	17.2 (13.8; 21.2)
Any breastfeeding at 3 months					
No	19.7 (17.0; 22.8)	35.2 (27.8; 43.4)	9.1 (5.4; 15.1)	23.2 (17.0; 30.9)	19.0 (13.4; 26.3)
Yes	80.3 (77.2; 83.0)	32.4 (28.7; 36.3)	10.9 (8.6; 13.7)	23.7 (20.4; 27.4)	17.2 (14.3; 20.5)
Any breastfeeding at 6 months					
No	34.6 (31.1; 38.2)	29.5 (24.0; 35.5)	6.2 (3.8; 10.7)	19.9 (15.3; 25.4)	14.9 (11.0; 20.0)
Yes	65.4 (61.8; 68.9)	34.0 (29.8; 38.5)	12.7 (10.0; 16.1)	25.4 (21.6; 29.6)	19.1 (15.7; 22.9)
Any breastfeeding at 12 months					
No	65.7 (62.2; 69.1)	32.8 (28.8; 37.2)	9.1 (6.9; 12.1)	22.0 (18.5; 26.0)	16.0 (13.0; 19.6)
Yes	34.3 (30.9; 37.8)	33.1 (27.5; 39.1)	13.5 (9.8; 18.4)	26.3 (21.2; 32.1)	21.1 (16.5; 26.6)
Sugar consumption at 12 months					
No	10.9 (8.7; 13.5)	29.6 (20.1; 41.2)	8.4 (3.8; 17.6)	21.1 (13.1; 32.2)	15.5 (8.8; 25.9)
Yes	89.1 (86.5; 91.3)	32.5 (28.8; 36.4)	10.5 (8.2; 13.3)	23.1 (19.8; 26.7)	16.9 (14.0; 20.1)
Sugar consumption at 24 months					
Low	52.2 (48.4; 55.9)	29.3 (24.8; 34.3)	8.3 (5.8; 11.7)	19.5 (15.7; 24.0)	14.1 (10.8; 19.1)
High	47.8 (44.0; 51.6)	36.0 (31.0; 41.5)	11.9 (8.8; 16.0)	27.3 (22.7; 32.4)	21.6 (17.4; 26.5)
	**Brazil (age 4 years)**				
	Overall	ECC present	SECC present	dmfs>0	d>0
	% (95% CI)	% (95% CI)	% (95% CI)	% (95% CI)	% (95% CI)
Exclusive breastfeeding at 3 months					
No	54.6 (53.0; 56.3)	35.8 (33.7; 38.0)	18.9 (17.2; 20.7)	23.9 (22.1; 25.9)	23.4 (21.6; 25.4)
Yes	45.4 (43.7; 47.0)	38.6 (36.2; 41.0)	23.4 (21.4; 25.6)	29.9 (27.7; 32.2)	28.8 (26.6; 31.1)
Exclusive breastfeeding at 6 months					
No	87.0 (85.8; 88.1)	38.0 (36.3; 39.7)	21.5 (20.0; 23.0)	26.9 (25.4; 28.6)	26.4 (24.8; 28.0)
Yes	13.0 (11.9; 14.1)	33.0 (28.8; 37.4)	18.7 (15.4; 22.5)	26.4 (22.5; 30.6)	24.2 (20.5; 28.3)
Any breastfeeding at 3 months					
No	22.2 (20.8; 23.6)	33.9 (30.7; 37.3)	16.6 (14.2; 19.4)	21.2 (18.5; 24.3)	20.7 (18.0; 23.7)
Yes	77.8 (76.4; 79.2)	38.0 (36.2; 39.8)	22.2 (20.7; 23.8)	28.2 (26.5; 29.9)	27.3 (25.7; 29.0)
Any breastfeeding at 6 months					
No	45.3 (43.7; 46.9)	32.6 (30.4; 35.0)	15.9 (14.2; 17.8)	20.2 (18.3; 22.2)	19.7 (17.8; 21.7)
Yes	54.7 (53.0; 56.3)	40.8 (38.6; 43.0)	25.2 (23.3; 27.2)	32.0 (30.0; 34.1)	30.9 (28.9; 33.0)
Any breastfeeding at 12 months					
No	57.2 (55.5; 58.8)	32.0 (30.0; 34.1)	15.5 (14.0; 17.2)	20.4 (18.7; 22.2)	19.7 (18.1; 21.5)
Yes	42.8 (41.2; 44.5)	43.8 (41.3; 46.3)	28.3 (26.0; 30.6)	35.0 (32.7; 37.5)	34.0 (31.6; 36.4)
Sugar consumption at 12 months					
No	48.2 (46.4; 49.9)	27.6 (25.4; 30.0)	13.1 (11.5; 14.9)	18.5 (16.6; 20.5)	17.6 (15.7; 19.6)
Yes	51.8 (50.1; 53.6)	44.2 (41.8; 46.6)	26.3 (24.2; 28.5)	32.0 (29.7; 34.3)	31.4 (29.2; 33.7)

Sugar consumption at 24 months					
Low	55.0 (53.4; 56.7)	30.4 (28.4; 32.5)	15.5 (13.9; 17.2)	20.6 (18.9; 22.5)	19.7 (18.0; 21.6)
High	45.0 (43.3; 46.6)	45.2 (42.8; 47.7)	27.6 (25.5; 29.9)	34.0 (31.7; 36.3)	33.2 (30.9; 35.6)

*Total number of children for each variable may differ since the sample comprises all children included in at least one of the sub-analyses. ECC: Early Childhood Caries. S-ECC: Severe Early Childhood Caries. dmfs: decayed, missing, or filled surfaces. d: decayed surfaces.

https://doi.org/10.3390/xxxxx

Supplementary Table S2 - Adjusted analysis for the controlled direct effect of not breastfeeding on dental caries based on Marginal structural models, and sensitivity analyses based on adjusted estimates.

		Australia Age 5 years			Brazil Age 4 years		
		aRR (95% CI)	E-value		aRR (95% CI)	E-value	
			aRR	CI		aRR	CI
Exposure	Not BF 3 months	n=631			n=3,081		
Mediator	Sugar 12 months						
ECC	CDE	1.05 (0.33; 3.35)	1.28	NA	0.77 (0.60; 0.98)	1.92	1.16
SECC	CDE	1.13 (0.59; 2.16)	1.51	NA	0.62 (0.40; 0.96)	2.61	1.25
dmfs>0	CDE	0.45 (0.06; 3.38)	3.87	NA	0.62 (0.43; 0.88)	2.61	1.53
d>0	CDE	0.89 (0.52; 1.53)	1.50	NA	0.62 (0.43; 0.88)	2.61	1.53
Exposure	Not BF 6 months	n=619			n=3,101		
Mediator	Sugar 12 months						
ECC	CDE	1.14 (0.34; 3.80)	1.54	NA	0.69 (0.57; 0.83)	2.26	1.70
SECC	CDE	1.95 (0.09; 3.88)	3.31	NA	0.56 (0.40; 0.77)	2.97	1.92
dmfs>0	CDE	1.22 (0.30; 4.98)	1.74	NA	0.55 (0.42; 0.71)	3.04	2.17
d>0	CDE	0.26 (0.03; 2.28)	7.15	NA	0.54 (0.42; 0.71)	3.11	2.17
Exposure	Not BF 12 months	n=667			n=3,503		
Mediator	Sugar 24 months						
ECC	CDE	1.16 (0.79; 1.70)	1.59	NA	0.63 (0.55; 0.72)	2.55	2.12
SECC	CDE	1.37 (0.59; 3.19)	2.08	NA	0.43 (0.34; 0.55)	4.08	3.04
dmfs>0	CDE	0.99 (0.61; 1.60)	1.11	NA	0.50 (0.42; 0.61)	3.41	2.66
d>0	CDE	0.76 (0.43; 1.35)	1.96	NA	0.49 (0.40; 0.60)	3.50	2.72

*Adjusted for maternal education, household income, partnership, maternal age, parity, and age at dental examination, and including an interaction term between exposure and mediator.

BF: Breastfeeding. RR: Relative Risk. 95% CI: 95% confidence interval. ECC: Early Childhood Caries. S-ECC: Severe Early Childhood Caries. dmfs: decayed, missing, or filled surfaces. d: decayed surfaces. CDE: Controlled direct effect.

Supplementary Table S3. Crude and adjusted analysis for the controlled direct effect of not exclusively breastfeeding on dental caries, and sensitivity analyses based on adjusted estimates. Marginal structural models. 2015 Pelotas Birth Cohort Study.

		Australia Age 5 years			
		Crude RR[1] (95% CI)	Adjusted RR[2] (95% CI)	E-value	
Exposure	Not Exclusive BF 3 months	n=631	n=631	aRR	CI
Mediator	Sugar 12 months				
ECC	CDE	2.06 (0.91; 4.65)	1.91 (0.75; 4.87)	3.23	NA
SECC	CDE	3.91 (0.43; 35.60)	5.82 (0.62; 54.67)	11.12	NA
dmfs>0	CDE	1.84 (0.65; 5.22)	1.70 (0.54; 5.40)	2.79	NA
d>0	CDE	1.30 (0.35; 4.76)	0.94 (0.22; 3.93)	1.32	NA
		Brazil Age 4 years			
		Crude RR[1] (95% CI)	Adjusted RR[2] (95% CI)	E-value	
Exposure	Not Exclusive BF 3 months	n=3,075	n=3,075	aRR	CI
Mediator	Sugar 12 months				
ECC	CDE	0.77 (0.66; 0.91)	0.74 (0.61; 0.89)	2.04	1.50
SECC	CDE	0.63 (0.48; 0.82)	0.64 (0.47; 0.86)	2.50	1.60
dmfs>0	CDE	0.61 (0.49; 0.76)	0.60 (0.47; 0.77)	2.72	1.92
d>0	CDE	0.61 (0.48; 0.76)	0.60 (0.47; 0.78)	2.72	1.88
Exposure	Not Exclusive BF 6 months	n=3,062	n=3,062	E-value	
Mediator	Sugar 12 months			aRR	CI
ECC	CDE	0.97 (0.78; 1.21)	0.72 (0.56; 0.93)	2.12	1.36
SECC	CDE	0.99 (0.70; 1.40)	0.70 (0.45; 1.10)	2.21	NA
dmfs>0	CDE	0.85 (0.65; 1.11)	0.56 (0.41; 0.77)	2.97	1.92
d>0	CDE	0.91 (0.68; 1.20)	0.58 (0.42; 0.81)	2.84	1.77

[1]Adjusted for age at dental examination and including an interaction term between exposure and mediator.
[2]Adjusted for age at dental examination, maternal education, household income, partnership, maternal age, parity, and including an interaction term between exposure and mediator.
BF: Breastfeeding. RR: Relative Risk. 95% CI: 95% confidence interval. ECC: Early Childhood Caries. S-ECC: Severe Early Childhood Caries. dmfs: decayed, missing, or filled surfaces. d: decayed surfaces. CDE: Controlled direct effect.
